# A cross-sectional study examining the role of doctors’ trust in patients’ requests for antibiotics: a neglected perspective

**DOI:** 10.1186/s13690-025-01677-2

**Published:** 2025-07-17

**Authors:** Aline Rinaldi, Serena Petrocchi, Anna Bullo, Luca Gabutti, Peter Johannes Schulz

**Affiliations:** 1 Faculty of Communication, Culture and Society, Università della Svizzera Italiana, Lugano, Switzerland; 2 Faculty of Biomedical Sciences, Università della Svizzera Italiana, Institute of Family Medicine , Lugano, Switzerland; 3 Faculty of Biomedical Sciences, Università della Svizzera Italiana, Lugano, Switzerland; 4 Wee Kim Wee School of Communication and Information & LKC School of Medicine, Nanyang Technological University, Singapore, Singapore

**Keywords:** Patient-provider communication, Trust, Antibiotic request, Antimicrobial resistance

## Abstract

**Background:**

Effective interaction between patients and providers is central to understanding communication mechanisms and health-related outcomes. Antibiotic requesting behavior and its predictors are a crucial topic in the context of antimicrobial resistance, a global health challenge that increases healthcare expenditure and negatively impacts patient outcomes. A major determinant of antibiotic misuse is rooted in primary care, where patients’ high expectations and requests, combined with doctors’ lack of assertiveness, lead to overprescription and overconsumption. Many studies report that request, as a communicative behavior, puts great pressure on doctors affecting their decision-making process. Trust is a critical aspect of this relationship that may influence both patients’ willingness to request antibiotics and doctors’ responses. This study examines how trust from the doctors’ perspective, trust from the patients’ perspective and the interaction between the two – while controlling for covariates – impact patients’ intention to ask for antibiotics, providing insights into the interpersonal dimensions that contributes to antibiotic prescribing practices.

**Method:**

A cross-sectional study with 8 family doctors and 101 patients. The data gathering was performed from May to July 2024 in the Italian-speaking region of Switzerland. Post-visit questionnaires assessed trust from both parties, patients’ concerns, perceived susceptibility to illness, and symptom severity. Generalized Estimating Equations (GEE) accounted for data clustering.

**Results:**

Doctors’ trust in their patients significantly reduced patients’ intentions to request antibiotics (*p* =.02), even when controlling for covariates. Interaction effects revealed the moderating role of doctors’ trust in shaping patients’ antibiotics requests.

**Discussion:**

The results of this study highlighted the impact of doctors’ ratings of trust on patients’ intention to request antibiotics. Enhancing mutual trust in doctor-patient relationships could help reduce patient-driven antibiotic overprescription, providing a promising avenue for interventions addressing antimicrobial resistance.

**Supplementary Information:**

The online version contains supplementary material available at 10.1186/s13690-025-01677-2.

**Table:** 

Text box 1. Contributions to the literature
• Based on the literature, antimicrobial resistance and the misuse of antibiotics are global concerns that need to be addressed extensively. Our study contributes to existing literature by examining patients’ requesting behavior for antibiotics.
• The existing research lacks attention to the dyadic phenomena that arise in the patient-provider interaction and influence it. This study fills that gap by considering both patients’ and doctors’ perspectives.
• The method of Generalized Estimating Equation allowed for a dyadic analysis that also accounted for the non-independence of data and the clustering within 8 doctors.
• Findings suggest that doctors’ perceived trust in their patients may moderate patients’ willingness to request antibiotics.

## Background

The world is facing the hidden healthcare crisis of antimicrobial resistance (AMR) driven mostly by misuse of antibiotics in the outpatient sector, and leading to major consequences such as worsening health, longer hospital stays and increasing healthcare costs [1, 2]. Over the next two decades AMR could result in a sharp increase in mortality rates, potentially causing up to 10 million deaths per year, with associated costs rising proportionally [3]. The alarming rise in fatalities would primarily be driven by the growing ineffectiveness of antibiotics and other antimicrobial agents against bacteria that were once easily treatable, leading to more severe and prolonged infections [3]. The phenomenon is evolving on a global scale due to the continued emergence of strains of resistant bacteria and the interconnectedness of our world [4–7].

The issue of AMR is deeply rooted in the primary care sector, which is estimated to account for 80–90% of prescriptions for antibiotics [2, 8]. Various elements in family practice contribute to the misuse and overprescription of antibiotics. The main driving factors are diagnostic uncertainty, time constraints combined with the high numbers of patients presenting each day, and doctors’ cautious attitude toward taking risks with patients’ health. Rezal et al.’s systematic review [9] also discussed several factors that influence doctors’ prescribing behavior, including patients’ expectations, severity, and duration of infections, uncertainty over diagnosis, and doctors’ concern about potentially losing patients. Another leading factor is the pressure exerted by patients, who expect to receive antibiotics and directly request prescriptions [2]. A systematic review [10] also reports, among other factors, economic aspects (e.g., incentives) driving doctors’ intention to prescribe an antibiotic. Another study [11] underscores the role of doctors’ fear of losing their patients and consequently their income. Other studies [12, 13] have a focus on the legal aspects and the lack of regulations or homogeneous and stricter rules on antibiotic usage.

### The role of patient-provider interactions

As reported by the Economic and Social Research Council (ESRC) in a brief compiled in 2016 [14], antibiotic prescribing practices are deeply intertwined with the dynamics of the doctor-patient relationship. Trust, open communication, and continuity of care are central to this dynamic. A recent systematic review [15] demonstrated that in the discussion about antibiotic overprescription, communication and patient-provider behavior hold a central position. Other studies emphasize the contributing role of patients’ requests to the emergence of AMR along with more practical issues, such as scarcity of time and lack of resources for doctors to communicate effectively with their patients [16–18].

A qualitative study [19] found that patients’ willingness to seek antibiotics is shaped by their perceived symptom burden and beliefs about antibiotic effectiveness.

As for the consequences, patient demand has been consistently linked to higher chances of receiving a prescription, a pattern documented in several studies and systematic reviews [9, 20–24]. This correlation between patient demand and antibiotic prescription highlights a significant challenge in managing appropriate antibiotic use in healthcare.

Other studies [23–29] underscored the importance of the interpersonal dimension in the context of antibiotic prescription, indicating that general practitioners frequently encounter pressure from patients’ in the form of requests and expectations, even when such requests are not deemed appropriate or essential to the healing process. That is especially the case when confronted with respiratory tract infections (RTI), even though antibiotics are proven to have a limited impact against them [19, 20].

### The importance of trust in the healthcare context

Patient-physician trust plays a stabilizing role in shaping the dynamics and quality of the healthcare experience [30–33]. Moreover, the doctor-patient relationship is often characterized by an inherent imbalance, as patients are typically the most vulnerable party [34] due to several key factors: an information asymmetry, in which physicians possess specialized knowledge that patients lack; the authoritative role of the physician; and the emotional and physical vulnerability of patients who typically seek care during illness or distress [31, 32].

Trust is crucial in the patient-physician relationship, influencing various aspects of healthcare delivery, from communication and shared decision-making to patient adherence and satisfaction [35, 36]. Multiple studies reported on the positive outcomes of high trust, such as the formation of positive attitudes towards treatment and adherence, reduced pain perception, greater openness to sharing sensitive information and lower inclination to seek second opinions [37–41]. Patients with low trust in their physicians often feel less satisfied with their care, are less likely to follow medical advice and tend to report fewer improvements in their symptoms [40, 42].

Although trust in physicians is typically associated with greater adherence to medical advice and thus a lower likelihood of antibiotic misuse, trust may also shape patients’ expectations for antibiotic treatment. It is important to distinguish between these two constructs: misuse generally occurs after a prescription has been given, while expectations or intentions to request antibiotics arise before or during the consultation. In this light, trust may play a dual role. On one hand, it can reduce misuse by encouraging adherence to prescribed treatment. On the other hand, it may increase demand for antibiotics by fostering greater openness and willingness to communicate with the doctor, potentially leading to more direct requests, whether or not these requests are ultimately fulfilled. Relatively few studies have explored how trust shapes patients’ expectations for drugs. For example, one study [42] found that patients with higher levels of trust were more likely to request new medications in general, suggesting that a strong patient-provider relationship fosters openness and a greater willingness to express treatment preferences. Thorpe et al. [25] investigated how trust in physicians interacts with the information provided during consultations to shape patients’ expectations for antibiotics. Their findings suggest that trust can have a twofold influence. When patients highly trust their doctor but receive limited information about why antibiotics are unnecessary, they are more likely to hold inappropriate expectations for antibiotic treatment. In contrast, when clear and detailed explanations are given, trust contributes to lowering those expectations. Results of this study suggest that the relationship between patients’ trust and their expectation of receiving antibiotics might be moderated by other factors, highlighting a dynamic worthy of further investigation.

Physicians’ trust in patients may also shape how patients communicate their treatment preferences, including requests for unnecessary medications. Although physicians’ trust and patients’ behavior pertain to two different individuals, the relational dynamics of the clinical encounter suggest that they are closely intertwined. Patients are highly sensitive to how they are perceived by their doctors, and subtle cues of trust or mistrust can influence their attitudes and choices during the consultation. This mechanism works through an interpretation of affective cues, which is informed by prior experiences, expectations, and the evolving dynamics of the clinical interaction [43]. As in any interpersonal exchange, the emotional atmosphere is co-constructed and plays a key role in shaping mutual perceptions and behaviors. Patients may interpret a physician’s trust not only through explicit verbal statements, but also through non-verbal communication such as tone of voice, body language, attentiveness, and expressions of empathy. These cues contribute to an overall emotional climate that patients interpret as either validating or distancing [44]. When patients perceive a lack of trust, they may feel compelled to assert themselves more forcefully, for instance, by insisting on specific treatments like antibiotics. In contrast, feeling trusted and respected can encourage patients to adopt a more collaborative attitude and rely more on the physician’s clinical judgment.

### A dyadic approach to consider doctor-patient relationships

Given the intricate nature of doctor-patient relationships, a dyadic framework provides a more comprehensive understanding of how trust and behavior are mutually shaped, yet relatively few studies have employed a dyadic approach to communication and trust in healthcare.

In a study on doctor-patient communication [44, 45], it was explained that doctors and their patients have very different perspectives of doctors’ communication skills during routine clinical encounters. Another study [46] analysed doctor-patient trust, discovering a reciprocal effect of trust. Both studies applied a dyadic analysis approach called “One With Many”, where the physician acted as a focal person and common element across all participants. Such methodologies, which are designed to accommodate the hierarchical structure of the data, enhance the reliability of the findings by accurately reflecting the interdependencies inherent in the patient-physician dynamic [47].

No research applied a dyadic approach to the study of antibiotic requests from patients, stressing another gap in knowledge, especially from the analytical methodologies employed. Classic statistical analysis does not adequately address the clustering of patients under individual physicians, resulting in a critical oversight. By assuming the independence of the collected data, these analyses position physicians as passive recipients of influence from their patients, thereby failing to capture the complexities of the dynamic, and misrepresenting the influences that shape both patient and physician behaviors.

Some advancements toward this perspective have been made through the adoption of analytical methods such as Generalized Estimating Equations and mixed logit models, which account for the clustering of patients under different physicians [48, 49]. This evolution in analytical approaches indicates a shift in conceptualization towards a more interpersonal understanding of the dynamics at play. Therefore, this study specifically applies a dyadic conceptual level of analysis, considering both patients’ and doctors’ views and applying a corresponding dyadic level of analysis to account for the interdependence of the data.

### The aims of the study

The aim of the study is to investigate the factors influencing patient’s intention to request antibiotics by adopting an interpersonal approach and introducing in the analysis the reciprocal ratings of trust from both doctors and patients.

As a first step we analyzed the relational construct of patients’ trust in physicians and its role regarding their intention to ask for antibiotics. As underscored in the introduction, studies performed on patients’ trust generated mixed results which prevented us from formulating a specific hypothesis, but rather generates the following research question:

#### RQ1

How does patient’s trust in the doctor influences the intention to ask for antibiotics?

Secondly, we moved from the individual patient-level to consider the interdependence of the patient-provider relationship. To bridge the gaps in research for what concerns the role of doctors’ trust, we investigated how doctors’ trust in their patients influences patients’ requests for antibiotics in a hypothetical scenario. The lack of prior research on this topic prevents us again from the formulation of a specific hypothesis, leading instead to a second research question:

#### RQ2

How does the doctors’ trust in patients influences patients’ intention to ask for antibiotics?

Consequently, we tested the interactions between the two ratings to gain more knowledge on how both perspectives can influence patients’ willingness to request antibiotics in a hypothetical scenario. This is also an innovative aspect of our research for which previous studies are lacking, some dyadic studies [41] performed on different contexts allow us to hypothesize that the interactions between patient and physicians’ trust ratings will have a significant effect, but the lack of specific research on the topic leads us to formulate a third research question:

#### RQ3

How does the interaction between patient’s and doctors’ trust affect patients’ intention to ask for antibiotics?

The abovementioned relationships have been controlled for several patients’ characteristics, such as socio-demographic variables, and susceptibility to illness, perceived severity of symptoms, concern related to health problems, these constructs have been derived from the Health Belief Model (HBM) [50]. Moreover, these same constructs have been considered in a moderation analysis of the relationship between trust and intention to request antibiotics.

**Fig. 1:**
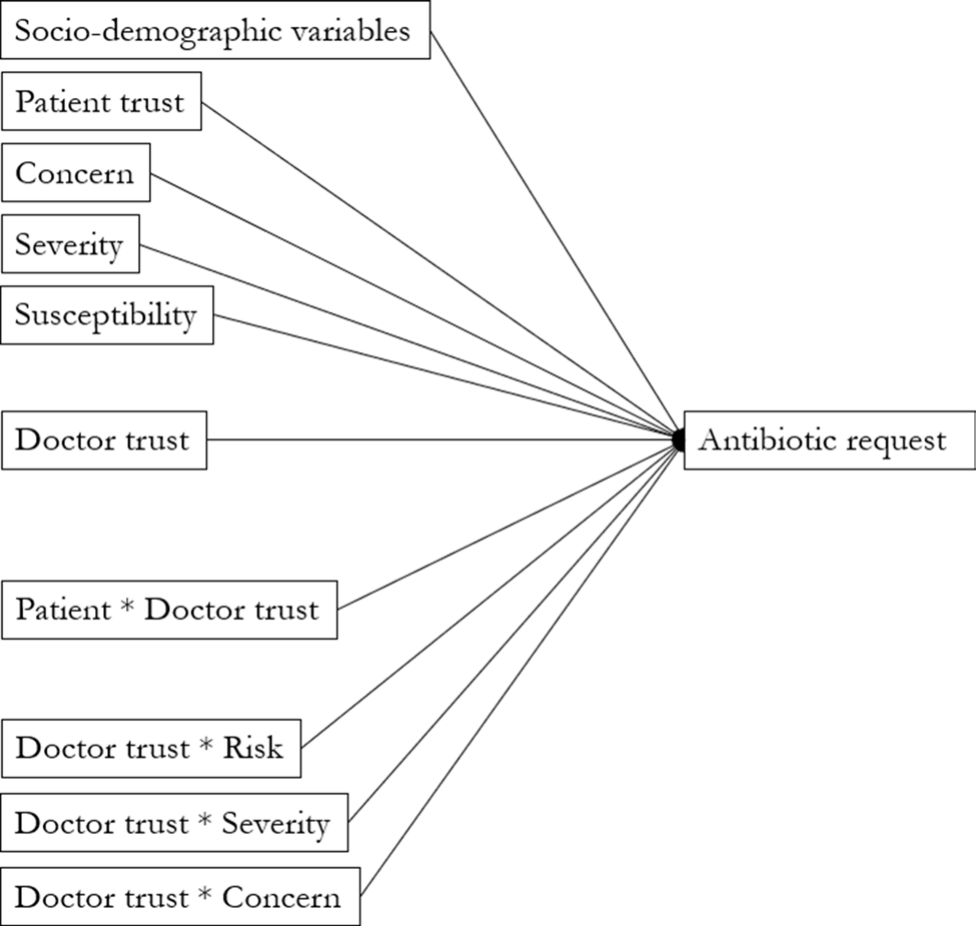
Visualization of the conceptual framework of the study

A visualization of the conceptual framework is presented in Fig. 1.

## Method

### Procedure

A cross-sectional study was conducted using questionnaires. The data-gathering process started in May 2024 and lasted until July of the same year. The selection of participants was carried out in two phases. In the first phase, family doctors were contacted and given the opportunity to participate in the study; during the second phase, the selection of patients was on a voluntary basis, and it took place in the doctors’ offices. Each doctor who agreed to participate was expected to indicate a date for the data gathering when a researcher could stay in the waiting room. The recruitment was performed by the doctor after each visit with a patient. When a patient accepted to participate, they were directed towards the researcher to sign the informed consent and fill in a questionnaire. A list of family doctors was compiled by confronting regional search engines and official cantonal lists of practicing physicians. We were able to find contact information for 48 doctors, who were contacted by email or by phone, and given preliminary information on the study; 8 accepted to participate, with an acceptance rate of 17%. Patients eligible to participate needed to be at least 18 years old, be Swiss residents, and be able to speak and understand Italian. Patients were visiting for various reasons, and none had requested or been prescribed an antibiotic.

The study was based on two post-visit questionnaires, one completed by doctors and one by each patient recruited. The first part of the patient’s questionnaire gathered sociodemographic data; the second part evaluated their perception of susceptibility to illness, severity of symptoms, and concern about specific recurrent or seasonal illnesses for which antibiotic requests are common [5, 51, 52]. Patients were also asked to rate their trust in the attending physician and their intention to ask for an antibiotic for the same common illnesses. The intention to ask for antibiotics was assessed through a hypothetical question and did not refer to the specific consultation. As such, it captured a more general predisposition or intention rather than the participants’ actual request of antibiotics during the visit.

Doctors were asked to complete their socio-demographic information during the first encounter with the researchers. After each visit with a recruited patient, a questionnaire was administered to doctors with a measure of trust toward each patient.

The relevant ethical committee approved the research (2022 − 01965, Rif CE TI 4225).

### Participants

Eight family doctors agreed to participate in the study and a total of 101 patients were involved, with patient numbers per doctor ranging from 4 to 19 patients. The doctors were equally divided into females and males and had a mean age of 52.88 years (SD = 11.64), with the youngest doctor being 34 years old and the oldest being 68 years old. Most physicians were married (5 out of 8, 62.5%). Doctors had been practicing on average for 8.88 years (SD = 5.22 years). The physician with the least experience in the field reported having practiced for two years, while the one with the longest career 15 years. Socio-demographic information on the doctors is reported in Table 1.

**Table 1:** Socio-demographic information of family Doctors (*N* = 8) recruited from May to July of 2024 in the Italian speaking region of Switzerland to study the role of Doctors’ trust on patients’ intention to ask for antibiotics. The table reports, for each variable, the possible values (i.e. Items), mean, standard deviation (SD), minimum and maximum values of the variable (i.e. Min-Max), and percentage distribution, according to the level of the measurement of the variable

Variable	Items	Mean (SD)	Min-Max	Percentage
Gender	Female			50%
	Male			50%
	Other			0%
Age (years)		52.88 (11.64)	34–68	-
Civil status	Single			12.5%
	Married			62.5%
	Divorced			25%
	Widow			0%
Years of experience as a family doctor		8.88 (5.22)	2–15	-

A slight majority of patients were male (54.1%), and the average age was 63.67 years (SD = 15.73), with ages ranging from 21 to 19 years. Most patients reported having a stable partner (53.6%), and the majority reported high school diploma as their highest level of education (46.9%). Regarding their health status, 38.1% of patients reported having an average level of health, while 37.1% indicated being in good health. More information on patients is reported in Table 2.

**Table 2:** Socio-demographic information of the patients (*N* = 101) recruited from May to July of 2024 in the Italian speaking region of Switzerland to study the role of doctors’ trust on patients’ intention to ask for antibiotics. The table reports, for each variable, the possible values (i.e., Items), mean, standard deviation (SD), minimum and maximum values of the variable (i.e., Min-Max), and percentage distribution, according to the level of the measurement of the variable

Variable	Items	Mean (SD)	Min-Max	Percentage
Gender	Female			45.9%
	Male			54.1%
	Other			0%
Age (years)		63.67 (15.73)	21–91	
Education level	Compulsory education			11.5%
	Professional training			24%
	High School			46.9%
	Bachelor’s degree			8.3%
	Higher degree			9.4%
Civil status	Single			46.4%
	With a stable partner			53.6%
Perceived health status	Extremely poor			1%
	Poor			13.4%
	Average			38.1%
	Good			37.1%
	Extremely good			10.3%
Chronic illnesses	No			57.9%
	Yes			42.1%

### Measures

#### Patients’ measures

Patients were asked to provide socio-demographic information, such as their age, their marital status, and their highest level of education. Additionally, they were asked to rate their health status on a five-point Likert scale, ranging from “very poor” (1) to “very good” (5), and whether they had a chronic illness (yes/no). They were also asked to answer a set of questions. Table 3 reports the items for each measure, score ranges, skewness and kurtosis, and reliability coefficients.

**Table 3:** Scales description. Per each scale, the items are reported and then the descriptives with: number of responses (N), minimum-maximum values, mean, standard deviation (SD), variance, normal distribution indicators (skewness and kurtosis), and measures of consistency (i.e., Cronbach’s alpha and McDonald’s omega). The scale were used to study doctors’ trust and the influence on patients’ intention to request antibiotics. Data were gathered from May to July of 2024 in the Italian speaking region of Switzerland

Variable	Items	*N*	Min-Max	Mean (SD)	Variance	Skewness (SE)	Kurtosis (SE)	Cronbach’s alpha α (McDonald’s omega ω)
Patient perception of susceptibility to illness	How would you rate your risk of getting 1. a cold 2. the flu 3. a urinary infection 4. a respiratory infection	98	0–8	2.92 (1.94)	3.77	0.43 (0.24)	− 0.44 (0.48)	0.773 (0.775)
Patient perception of concern	How worried would you be if you got… 1. a cold 2. the flu 3. a urinary infection 4. a respiratory infection	98	1–5	2.52 (0.96)	92	0.44 (0.24)	− 0.07 (0.48)	0.841 (0.819)
Patient perception of the severity of illness	Factor 1 How would you rate the severity of your symptoms when you get 1. a cold 2. the flu	98	1–5	2.28 (0.97)	0.94	0.28 (0.24)	− 0.41 (0.48)	0.845
	Factor 2 How would you rate the severity of your symptoms when you get 1. a urinary infection 2. a respiratory infection	98	1–5	2.32 (0.89)	0.79	0.04 (0.24)	− 0.39 (0.48)	0.646
Patient’s trust in the doctor	“My doctor is extremely meticulous and attentive” “I completely trust my doctor’s decisions” “My doctor is very honest in presenting the different treatment options to me” “Overall, I have a lot of trust in my doctor”	98	2.75-5	4.72 (0.50)	0.25	-2.14 (0.24)	4.49 (0.48)	0.871 (0.877)
Patient intention to ask for an antibiotic	How likely are you to ask for an antibiotic in case you get 1. a cold 2. the flu 3. an urinary infection 4. a respiratory infection	98	1–5	2.36 (1.06)	1.12	0.52 (0.24)	− 0.81 (0.48)	0.846 (0.822)
Doctor’s trust in the patient	Factor 1: How confident are you that… 1. the patient has provided all the necessary medical information 2. the patient will inform you when there are substantial changes in their condition 3. the patient has communicated all the medications and therapies they are using 4. the patient has understood what you communicated to them 5. the patient will follow the recommended treatment plan 6. the patient will actively participate in the management of their condition 7. the patient will inform you if they do not follow the treatment plan	101	2.14-5	4.38 (0.66)	0.44	-1.17 (0.24)	0.91(0.48)	0.923 (0.928)
	Factor 2: How confident are you that… 1. the patient will respect the time available for the visit 2. the patient will respect personal boundaries 3. the patient will not make unreasonable requests 4. the patient will not manipulate the visit for personal gain.	101	1.50-5	4.46 (0.76)	0.58	-1.61 (0.24)	2.08 (0.48)	0.860 (0.883)

The first questions were operationalized to represent the main constructs of the HBM [50] that might be associated to the intention to ask for antibiotics. The model posits that individuals’ likelihood of engaging in a health-related action is primarily determined by their perception of the susceptibility to illness, the perceived level of concern when sick and the perceived severity of symptoms.

##### Susceptibility

Based on the principles and measurements of the HBM patients were asked to rate their susceptibility to four common illnesses on a scale ranging from 0 to 10 as for the example: “Rate your perceived risk of getting *the cold* ”. The items were averaged to measure the construct, with higher scores, indicating higher perceived susceptibility, yielding good internal consistency with α = 0.77 and ω = 0.76.

##### Concern

Patients were asked to rate their concerns when getting sick with a common illness with a five-point Likert scale ranging from “not concerned at all” (1) to “extremely concerned” (5) as for the example: “Rate your level of concern when you get *a cold*”. The items showed internal consistency, with α = 0.84 and ω = 0.82. The construct was measured by averaging the items in a composite score, with higher scores indicating higher levels of concern.

##### Severity

Patients were asked to rate the severity of their symptoms when affected by some common illnesses on a five-point Likert scale ranging from “not at all severe” (1) to “extremely severe” (5) as for the example: “Rate the severity of your symptoms when you get *a cold*”. Two composite scores were created: one considering the severity of symptoms when affected by a cold and flu, the second measuring the severity when affected by a urinary or a respiratory infection. These scores were created according to a previous qualitative study conducted on the same topic on a sample taken from the general population [53].

##### Patient’s trust in Doctor

Patients were asked to rate their trust in their doctors via the Trust in Physician short-form scale [54], the full scale is reported in Table 3. All items were measured on a five-point Likert scale ranging from “I don’t agree at all” (1) to “I completely agree” (5). The first item was dropped from the final score because it inflated the consistency of the scale. The scale showed good internal consistency with α = 0.87 and a ω = 0.88.

**Intention to ask for antibiotics**. Patients were asked to rate their hypothetical intention to request antibiotics when faced with common illnesses with a five-point Likert scale ranging from “no intention at all” (1) to “very strong intention to ask” (5) as for the example: “Rate your intention to ask for an antibiotic when presenting with *a cold*”. The scale showed good internal consistency with α = 0.85 and ω = 0.82. The averaged composite score of the four items was used for analysis, with higher scores corresponding to higher intention to ask for antibiotics.

#### Physicians’ measures

##### Doctor’s trust in their patient

Doctors were asked to rate their trust after visiting each patient with the Physician Trust in the Patient scale [55]. The scale was composed of 12 items evaluated on a five-point Likert scale ranging from “I do not agree at all” (1) to “I strongly agree” (5). The scale was subjected to a factor analysis according to what was done by the authors of the original scale [55]. The first factor measures doctors’ trust based on their patients’ communication abilities, and the other evaluates doctors’ trust based on their patients’ respect for professional boundaries. More details on the factorial analysis can be found in the section dedicated to the preliminary analyses. Two composite scores have been calculated as the average of the corresponding items, with higher scores corresponding to higher levels of trust. Both factors showed good internal consistency, with α = 0.92 and ω = 0.93 (communication factor) and α = 0.86 and ω = 0.88 (respect for boundaries).

### Data analysis strategy

The eight doctors completed the questionnaires with no missing data in their responses. Patients provided 101 questionnaires; however, seven were excluded from the final analysis due to missing data on critical variables. Analyses were performed on a dataset that comprised 94 observations from patients, each paired with their respective trust ratings provided by the attending physician.

The measures were subjected to consistency analysis, and the doctors’ trust in their patient scale was subjected to exploratory factorial analysis and Varimax rotation.

A series of Generalized Estimating Equations (GEE) were employed using R for R Studio (R 4.4.1) and the Geepack package [56] to estimate the influence of socio-demographic variables, patient’s evaluation of trust in physician, patient’s level of perceived susceptibility to illness, concern and severity of symptoms and doctor’s rated trust in patients on patients’ intention to ask for antibiotics (The R code used for analysis is provided in the supplementary material).

Demographic variables were included as patient-level predictors. Patients’ intention to request antibiotics was included as a dependent variable and conceptualized at the individual patient level.

Our estimations for the main effect model are based on Eq. (1)^1^ and this was called the Main Model in the analyses:

1 $$\begin{gathered} paskin{t_i}{_j} = {\text{ }}{\beta _0}{\text{ }} + {\text{ }}{\beta _1}\left( {pag{e_{ij}}} \right){\text{ }} + {\text{ }}{\beta _2}\left( {pgende{r_{ij}}} \right){\text{ }} \hfill \\+ {\text{ }}{\beta _3}\left( {ped{u_{ij}}} \right){\text{ }} + {\text{ }}{\beta _5}\left( {phealt{h_{ij}}} \right){\text{ }} + {\text{ }}{\beta _6}\left( {pchroni{c_{ij}}} \right) \hfill \\+ {\text{ }}{\beta _7}\left( {concer{n_{ij}}} \right){\text{ }} + {\text{ }}{\beta _8}\left( {psuscpetibilit{y_{ij}}} \right){\text{ }} + {\text{ }}{\beta _9}\left( {psev{1_{ij}}} \right){\text{ }} \hfill \\+ {\text{ }}{\beta _{10}}\left( {psev{2_{ij}}} \right) + {\beta _{11}}\left( {ptrus{t_{ij}}} \right)\, + {\text{ }}{\beta _{12}}\left( {dtrust{1_{ij}}} \right){\text{ }} \hfill \\+ {\text{ }}{\beta _{13}}\left( {dtrust{2_{ij}}} \right){\text{ }} + {\text{ }}{ \epsilon _{ij}} \hfill \\ \end{gathered}$$

We also tested for the interaction between doctors’ trust and the variables measured at the patient level, specifically patient’s perception of susceptibility to illness (Interaction Model 1), the patient’s degree of concern when ill (Interaction Model 2), and the two factors for patient’s perceived severity of symptoms of cold/flu and urinary/respiratory infections (Interaction Model 3 and Interaction Model 4). Interactions were tested one by one as for the example reported in Eq. (2)^2^

2 $$\begin{gathered} paskin{t_{ij}} = {\text{ }}{\beta _0}{\text{ }} + {\text{ }}{\beta _1}\left( {pag{e_{ij}}} \right){\text{ }} + {\text{ }}{\beta _2}\left( {pgende{r_{ij}}} \right){\text{ }} \hfill \\+ {\text{ }}{\beta _3}\left( {ped{u_{ij}}} \right){\text{ }} + {\text{ }}{\beta _5}\left( {phealt{h_{ij}}} \right){\text{ }} + {\text{ }}{\beta _6}\left( {pchroni{c_{ij}}} \right) \hfill \\+ {\text{ }}{\beta _7}\left( {pconcer{n_{ij}}} \right){\text{ }} + {\text{ }}{\beta _8}\left( {psusceptibilt{y_{ij}}} \right){\text{ }} \hfill \\+ {\text{ }}{\beta _9}\left( {psev{1_{ij}}} \right){\text{ }} + {\text{ }}{\beta _{10}}\left( {psev{2_{ij}}} \right){\text{ }} + {\text{ }}{\beta _{11}}\left( {ptrus{t_{ij}}} \right){\text{ }} \hfill \\+ {\text{ }}{\beta _{12}}\left( {dtrust{1_{ij}}} \right){\text{ }} + {\text{ }}{\beta _{13}}\left( {dtrust{2_{ij}}} \right){\text{ }} \hfill \\+\varvec {{\beta _{14}}(dtrust{1_{ij}} \times psusceptibilit{y_{ij}}) }+ {\epsilon _{ij}} \hfill \\ \end{gathered}$$

This method allowed for robust results and controlled the grouping of patients under different physicians, acknowledging the data’s non-independence. The model was fitted using an exchangeable correlation structure to account for the clustering of patients within medical groups. In total, there were 8 clusters corresponding to the medical groups, with up to 19 patients per cluster.

Several fit indices were calculated. QIC (Quasi-likelihood under the Independence model Criterion) is a fit index used in the context of GEE to assess the goodness of fit for a statistical model [57]. It is particularly useful where traditional likelihood-based criteria may not be applicable, a lower QIC value indicates a better-fitting model [57].

Plots were chosen as the method for graphically representing the interactions. Due to the small number of participants, it was not possible to test for the simple effect analysis; therefore, interactions and their corresponding plots will be presented in a more descriptive manner.

## Results

### Preliminary analyses

The items used to measure doctors’ trust revealed a two-factor structure accounting for 67.96% of the variance, with individual contributions of 39.66% and 28.30%. The Kaiser-Meyer-Olkin (KMO) measure of sampling adequacy was 0.876, indicating that the sample size was sufficient for factor analysis. Bartlett’s Test of Sphericity was significant, χ²(approximate) = 928.92, *p* <.001, suggesting that the correlation matrix was suitable for factor analysis.

### Main results

Table 4 reports the main effects model and the interaction models.

**Table 4:** Results from generalized estimating equations regression on patients’ intention to request antibiotics, including predictors such as age, gender, education level, health status, chronic illness, perceived susceptibility, concern, severity, type of illness, trust in Doctors and patients, communication skills, respect for boundaries, and interaction effects

	Main effects model	Interaction model 1	Interaction model 2	Interaction model 3	Interaction model 4
Model fit indices: QIC (QICC)	90.8 (26.2)	91.0 (25.0)	89.3 (23.3)	88.4 (22.4)	85.7 (19.7)
Variable	Estimate (SE)	Estimate	Estimate	Estimate	Estimate
Constant	3.38* (1.42)	4.20** (1.52)	0.03 (2.20)	0.18 (2.44)	0.66 (1.43)
Age	-0.01 (0.01)	-0.00 (0.00)	-0.01 (0.00)	-0.01 (0.01)	-0.01 (0.00)
Gender Female	-0.43** (0.15)	-0.48** (0.16)	-0.39** (0.15)	-0.40** (0.15)	-0.43** (0.15)
Education (Reference category: compulsory school) Professional training High school Bachelor’s degree Higher degree	-0.88*** (0.22) -0.23 (0.15) -0.57* (0.028) -1.04*** (0.29)	-0.94*** (0.23) -0.34* (0.14) -0.75* (0.36) -1.07*** (0.27)	-0.79*** (0.21) -0.14 (0.14) -0.48 (0.28) -0.94*** (0.27)	-0.76*** (0.19) − 0.23 (0.13) -0.48* (0.21) -1.06*** (0.30)	-0.76*** (0.19) -0.30** (0.10) -0.48* (0.21) -1.22*** (0.32)
Health status (Reference category: very poor) Poor Average Good Very good	1.69*** (0.39) 2.28*** (0.54) 2.39*** (0.52) 2.44** (0.76)	1.83*** (0.41) 2.38*** (0.54) 2.49*** (0.52) 2.63*** (0.77)	2.73*** (0.57) 3.20*** (0.69) 3.31*** (0.69) 3.29*** (0.81)	3.13*** (0.57) 3.62*** (0.67) 3.68*** (0.67) 3.69*** (0.74)	1.43*** (0.34) 2.07*** (0.43) 2.08*** (0.40) 2.12*** (0.61)
Chronic illnesses Yes	0.32 (0.23)	0.36 (0.26)	0.26 (0.20)	0.24 (0.20)	0.29 (0.20)
Concern	0.38** (0.12)	0.40*** (0.12)	1.31** (0.44)	0.38** (0.12)	0.38** (0.12)
Susceptibility	0.23*** (0.04)	-0.36 (0.26)	0.22*** (0.04)	0.22*** (0.04)	0.23*** (0.04)
Severity perception Cold and flu Urinary and respiratory infection	0.28** (0.10) -0.40** (0.15)	0.27** (0.10) -0.40** (0.13)	0.33*** (0.10) -0.43*** (0.13)	1.07*** (0.29) -0.35 (0.19)	0.32** (0.11) 1.15 (0.63)
Patient trust	-0.33 (0.24)	-0.27 (0.26)	-0.36 (0.23)	-0.35 (0.23)	-0.43 (0.23)
Doctor trust Communication abilities Respect of boundaries	− 0.02 (0.22) -0.46* (0.19)	0.02 (0.22) -0.77** (0.30)	0.56* (0.26) -0.46* (0.19)	0.02 (0.20) -0.06 (0.22)	0.01 (0.22) 0.34 (0.26)
Interaction terms Dtrust2*Psusceptibility Dtrust1*Pconcern Dtrust2*Psev1 Dtrust2*Psev2		0.13* (0.06)	-0.22* (0.11)	-0.19* (0.09)	-0.36** (0.14)

Note: In Table 4 only models with a significant interaction are reported

significance levels: “*” p = < 0.05; “**” p = < 0.01; “***” p = < 0.001

Dtrust1: refers to the doctor rating of the first factor of the evaluation of trust, which concerns more the interactional e communicative aspects

Dtrust2: refers to the doctor rating of the second factor of the evaluation of trust, which concerns the respect of roles in the interaction

Pconcern: refers to the patient rating of how worried she / he would be when affected by an illness

Psusceptibility: refers to the rating of the patient of how much they think they are at risk of catching an illness

Psev1: refers to the patient rating of how severe their symptoms would be if they were affected by a cold or the flu

Psev2: refers to the patient rating of how severe their symptoms would be if they were affected by a urinary or respiratory infection

Model fit indices indicate that every interaction model is better fitting than the one reporting only the main effects (QIC = 90.8; QICC = 26.2). In terms of fit indices, the best model is the fourth one (QIC = 85.7; QICC = 19.7).

#### Main model

The analyses revealed several significant predictors of patients’ intention to ask for antibiotics. While included as a control variable, age did not show a significant effect. Gender, by contrast, emerged as a significant factor: being female was associated with a reduction in the intention to ask for antibiotics. Educational attainment also played a significant role, with professional training or with a bachelor’s or a higher academic degree being associated with a decreased intention to ask for antibiotics. Health status also showed a consistent relationship with the outcome, with a better health status associated with an increase in the intention to ask for antibiotics.

In line with the assumptions based on the HBM, the analysis further identified perceived susceptibility and concern as strong predictors of patients’ intention to request antibiotics. Higher perceived susceptibility was associated with a significant increase in intention to request antibiotics; similarly, higher levels of concern were positively related to the general intention to ask for antibiotics.

In examining the trust variables, we found mixed results. The two factors representing doctors’ trust in patients showed contrasting patterns. While the first factor, relating to the patient’s abilities to communicate, was not significantly associated with the patient’s intention to request antibiotics, the second factor, measuring doctor’s rating of patients’ respect for boundaries, was a significant predictor. On the other hand, patient trust in the doctor did not significantly predict antibiotic-seeking behavior.

Overall, the model was robust in adjusting for clustering effects and showed an acceptable fit, indicating that the factors included in the model adequately explain variation in the outcome. The exchangeable correlation structure effectively accounted for the clustered nature of the data, and the estimated scale parameters confirmed the appropriateness of the Gaussian family and identity link function used.

#### Interaction models

**Fig. 2:**
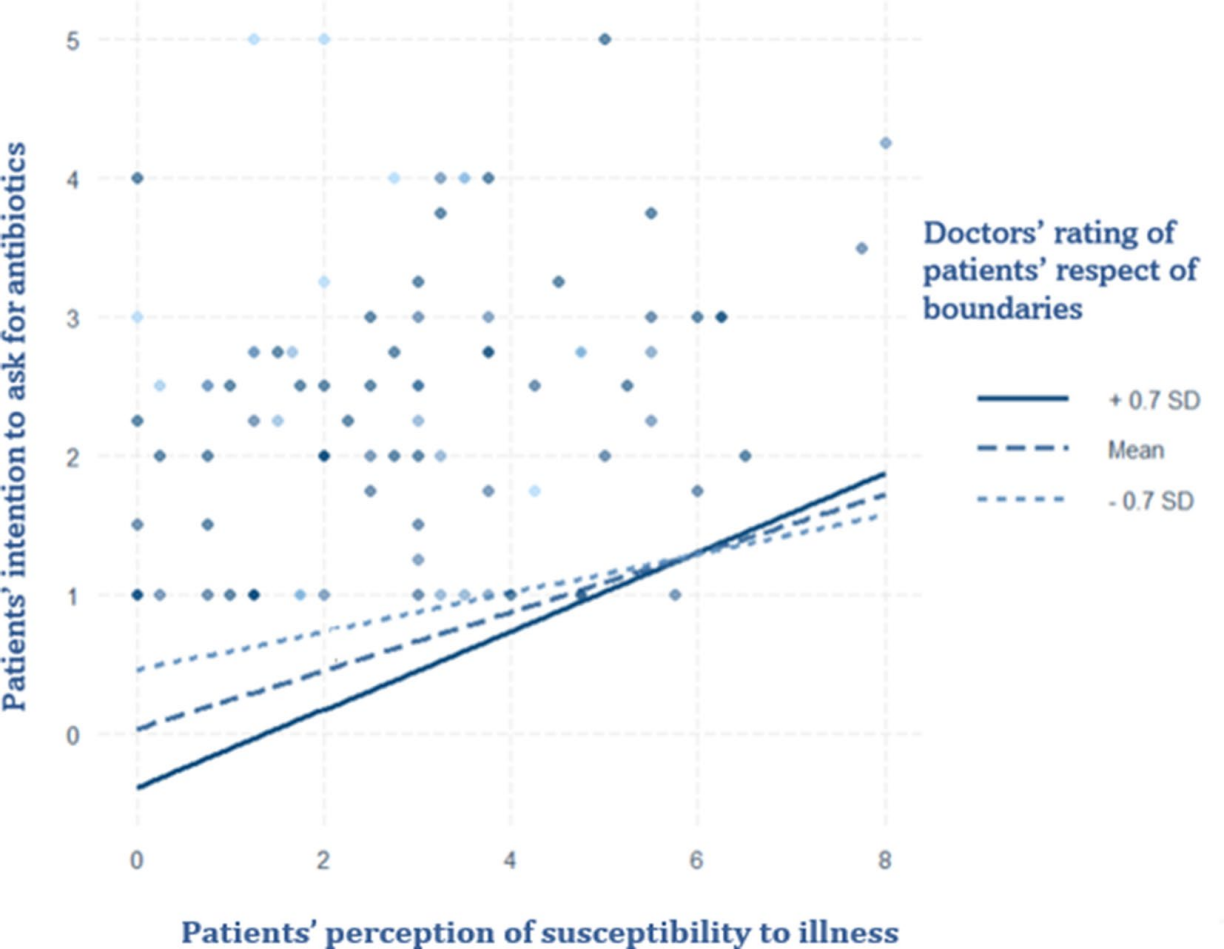
Interaction Plot of doctors’ rating of patients’ respect for boundaries and patients’ rating of susceptibility to illness on patient’s intention to ask for antibiotics. Data were gathered in the Italian speaking region of Switzerland from May to July 2024

Model 1 presents the results of the interaction between doctors’ rating of patients’ respect for professional boundaries and patients’ perception of their susceptibility to illnesses in predicting patients’ intention to request antibiotics. Compared with the main model, concern gained significance while susceptibility to illness became non-significant. The interaction is the only one that positively affected patients’ intention to ask for antibiotics. The corresponding plot (Fig. 2) shows that when doctors rate patients highly in terms of respect for professional boundaries, patients with a high perceived susceptibility to illness exhibit an increased intention to request antibiotics.

**Fig. 3:**
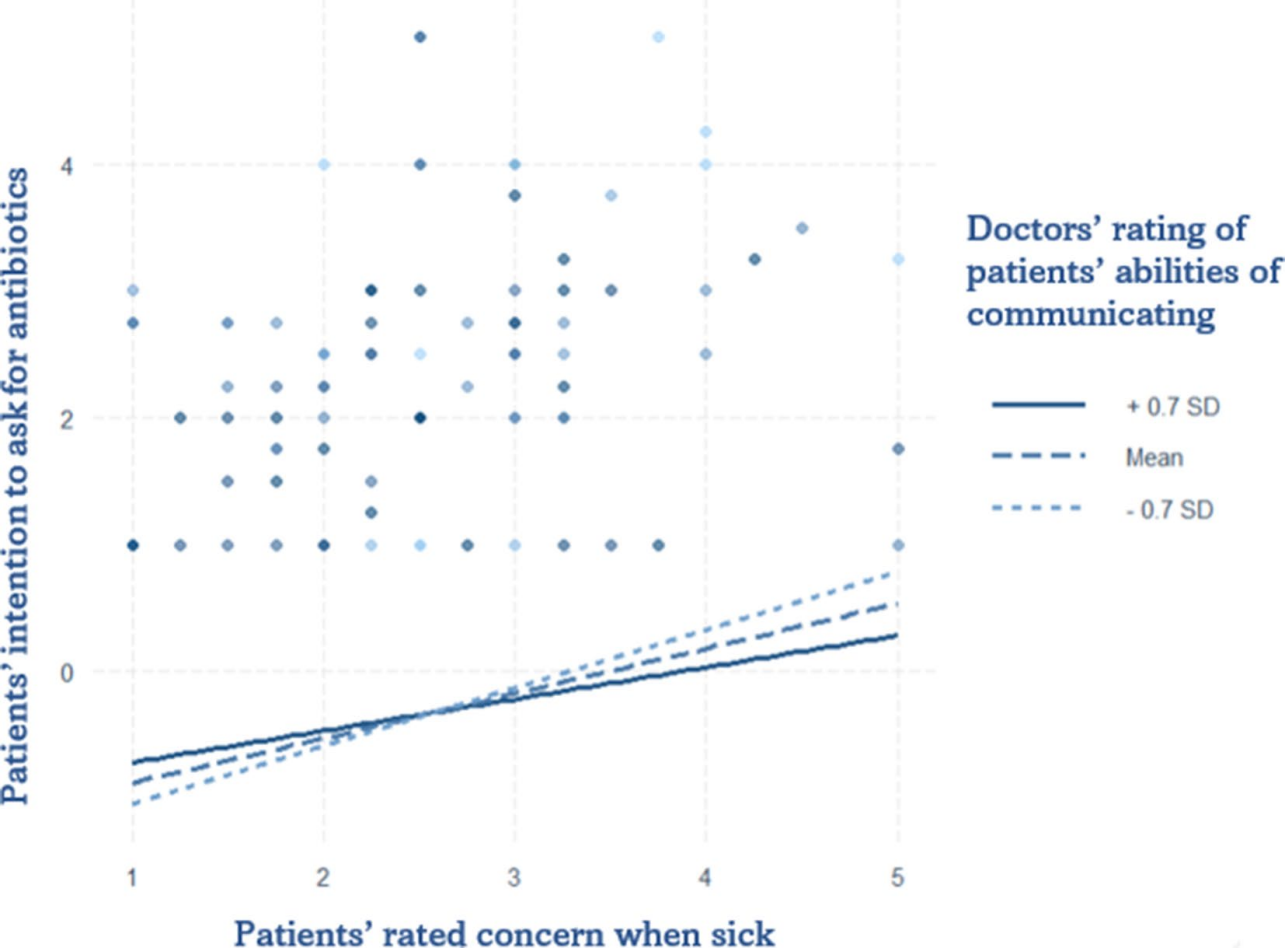
Interaction Plot of doctors’ trust level on patients’ communicating abilities and patients’ rating of concern levels when sick on patient’s intention to ask for antibiotics. Data were gathered in the Italian speaking region of Switzerland from May to July 2024

Model 2 presents the results of the interaction between the doctors’ rating of patients’ communication abilities and patients’ self-reported level of concern when they are sick. In comparison with the main model, the effects of perceived severity remained significant, while doctors’ rating of patients’ communication abilities became significant. The interaction reveals an inverse pattern, indicating a reduced intention to ask for antibiotics. The corresponding plot (Fig. 3) shows that the effect of a higher rating of trusting patients’ communication abilities from doctors corresponds to a lower intention from patients to ask for antibiotics even when they reported high levels of concern.

**Fig. 4:**
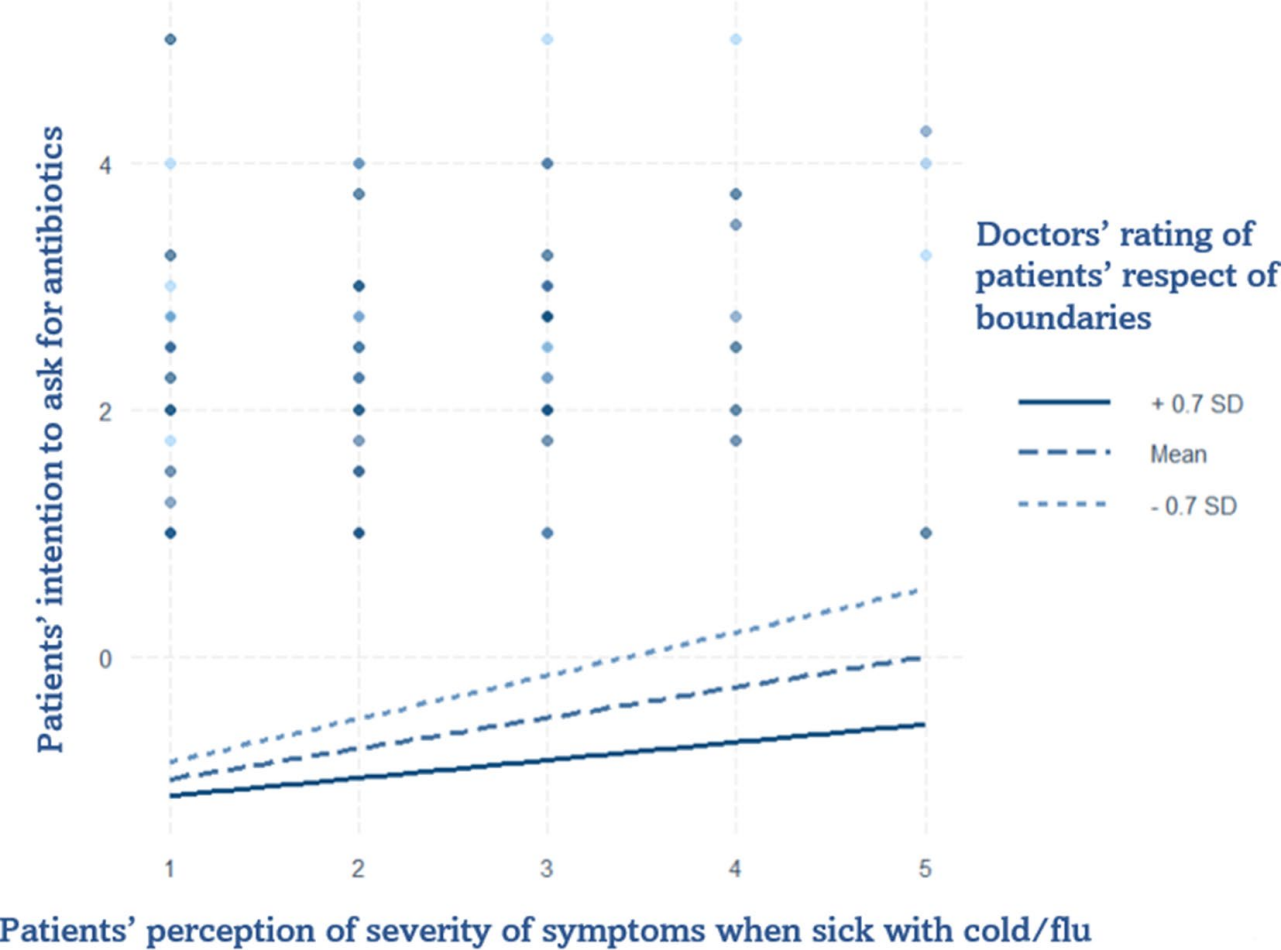
Interaction Plot of doctor’s rated respect for boundaries and patient’ rated severity of symptoms when sick with a cold or the flu on patient’s intention to ask for antibiotics. Data were gathered in the Italian speaking region of Switzerland from May to July 2024

Model 3 represents the interaction between doctors’ rating of patients’ respect for boundaries and patients’ perception of the severity of their symptoms when affected by the flu/cold. Comparing Model 3 with the main model, the severity of symptoms of cold/flu gained significance, while the severity of symptoms of urinary and respiratory infection lost significance, as did doctors’ rating of their patients’ respect for boundaries. The interaction showed a significant change in patients’ intention to ask for antibiotics with a decreasing trend. The plot (Fig. 4), indicates that higher perceived severity and higher evaluation of trust in the respect for boundaries, measured at the doctor level, corresponded a decrease in the patients’ intention to ask for antibiotics.

**Fig. 5:**
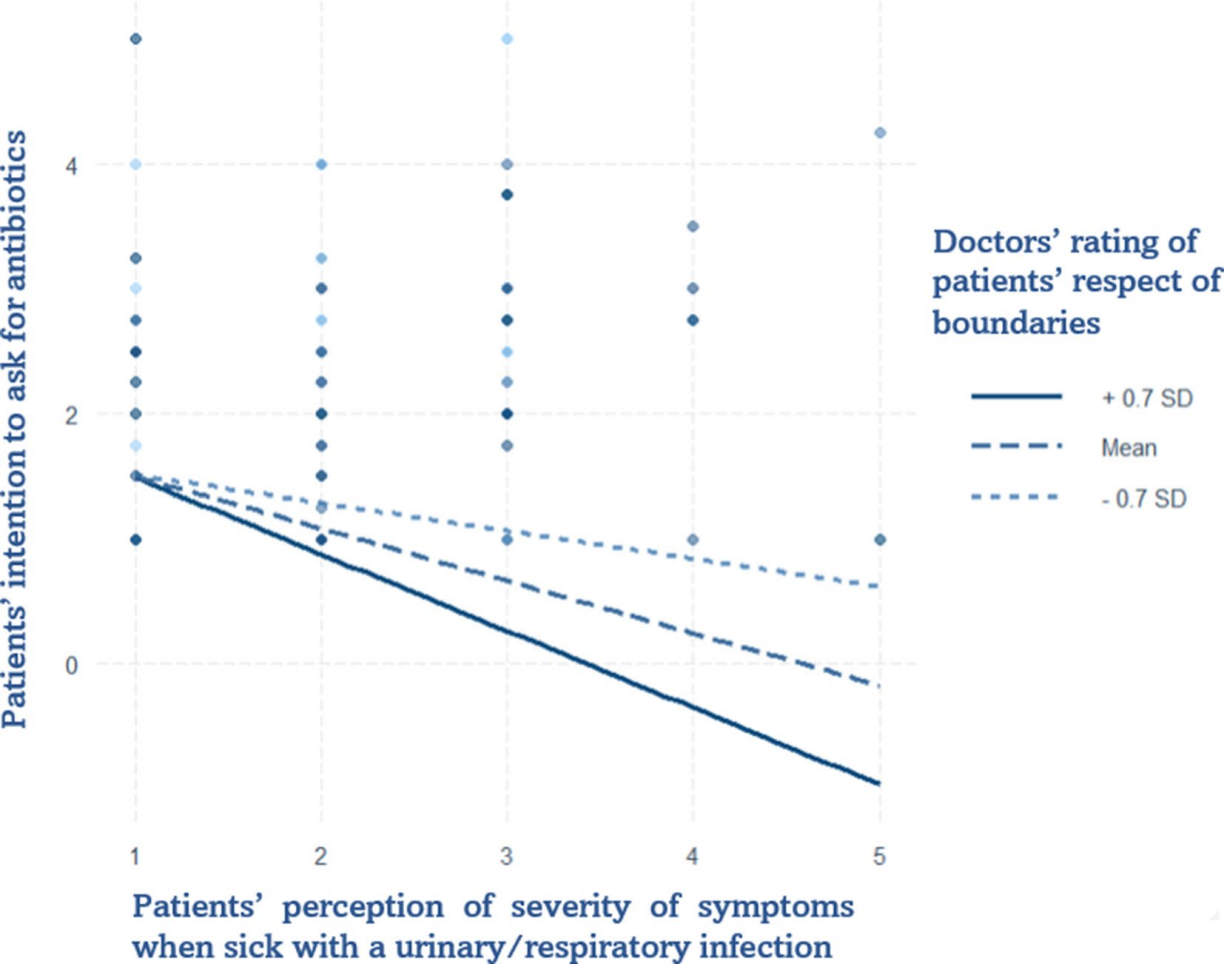
Interaction Plot of doctor’s rated respect for boundaries and patient’ rated severity of symptoms when sick with a urinary or a respiratory infection on patient’s intention to ask for antibiotics. Data were gathered in the Italian speaking region of Switzerland from May to July 2024

Model 4 shows the interaction between doctors’ trust that patients would respect professional boundaries and patients’ perception of the severity of their symptoms when affected by a urinary/respiratory infection. Compared to the main model, the severity of symptoms of urinary/respiratory infection lost its significance, as did doctors’ rating of patients’ respect for boundaries. The interaction was significant: the corresponding plot (Fig. 5) shows that when patients rate a higher severity of their symptoms, when the doctor rated a higher level of trust in the patient, the intention to ask for antibiotics significantly decreases.

## Discussion

This study investigated the complex dynamics of antibiotic requests within the doctor-patient relationship. Specifically, it examined the association between patients’ trust in their physician and their general intention to request antibiotics, as well as the role of physicians’ trust in their patients as a potential determinant of such intention. Furthermore, the study explored how physicians’ trust interacts with patients’ perceived susceptibility to illness, levels of concern, and perceptions of illness severity. The overarching aim was to provide a more nuanced understanding of the factors influencing patients’ intention to ask for antibiotics, contributing to the broader discourse on healthcare communication and prescribing practices.

As expected, findings indicate that patients who perceive themselves to be at higher risk of falling ill and perceive greater concern when ill have a stronger tendency to ask for antibiotics, further emphasizing the role of emotional responses in shaping patient decision-making regarding antibiotics [50].

Patients’ trust in their doctor did not emerge as a significant predictor of patients’ likelihood to request antibiotics. This result may be regarded as unexpected and suggests that other factors, such as patients’ perceptions of the severity of their illness or the level of worry associated with it, may take precedence over trust in guiding their decision-making processes. This finding contrasts with prior research [42], which indicated that patients who report higher trust in their physician are more inclined to request new medications. This discrepancy might be attributed to the dyadic evaluation approach employed in our study, which simultaneously considers the perspective of both the patient and the physician. This methodological framework enhances the understanding of the complex interplay between trust and medication-seeking behavior, by focusing on this dyadic evaluation.

At the same time, doctors’ trust that patients would respect professional boundaries significantly decreased patients’ intention to request antibiotics, while their ratings of trust that patients can communicate effectively did not have a significant result. The shift in patients’ intention might stem from greater confidence in their doctor’s recommendations or in the medical process.

The interaction models also offer interesting insights on the moderating role of doctors’ rated trust levels as they generally tend to reduce patients’ intention to request antibiotics, especially in cases where patients report high ratings of severity and concern. Of particular interest is the interaction with perceived severity of urinary or respiratory infections as they are medical conditions for which antibiotics are most commonly requested and prescribed [58, 59]. The significant interaction demonstrates that adherence to professional boundaries can result in a decreased intention to ask antibiotics. An unexpected result concerns the interaction with perceived susceptibility, as this is the only interaction that increases the intention to request antibiotics. We believe this trend may be due to the nature of susceptibility perception, as it evaluates the repeated possibility of contracting a specific illness over time, susceptibility is a construct that precedes illness and thus cannot be confined to a single episode, contrasting with concern and severity, which are likely assessed during the illness with a short-term perspective. We suggest that the increased intention to request antibiotics may be linked to this “long-term” and repeated perspective of the susceptibility assessment. Our results underscore the importance of trust from the physician’s perspective rather than the patient’s.

Interestingly, the physicians’ trust in their patients – specifically their rating of patients’ respect for boundaries – showed a significant effect. This trust directly influenced patients’ intentions to ask for antibiotics and acted as a moderating variable for other key patient-rated predictors, such as perceived susceptibility and severity, suggesting that the quality of the physician’s trust plays a pivotal role in shaping the interaction and contributes to whether patient concerns are validated or addressed. This study assumes that physicians’ trust in their patients, while measured from the doctors’ perspective, is not entirely internal or invisible during clinical encounters. We propose that patients are sensitive to a range of relational cues—such as tone of voice, eye contact, attentiveness, and emotional expression—that may signal how much the doctor trusts them [43]. These perceptions are likely shaped by prior experiences and expectations and can influence how patients choose to communicate during the consultation, including whether or not they feel the need to request antibiotics. This highlights the complexity of trust as a bidirectional dynamic, where the physician’s trust can significantly influence the patient’s behavior and overall consultation outcomes.

The doctors’ evaluation of patients’ respect for boundaries reflects the power imbalance and demonstrates its importance in medical interaction. In particular, the recognition and adherence to these professional boundaries appear to mitigate patients’ intention to request antibiotics. In the clinical setting, an unsolicited request for antibiotics may be perceived as overstepping upon the physician’s professional domain, given that the physician holds the responsibility for determining and prescribing appropriate antibiotic treatments.

The significant interactions observed with various dimensions of patient assessment imply a moderating role in the context of antibiotic requests.

### Limitations and recommendations for future studies

Our analyses allowed for some secondary results indicating that the intention to request antibiotics is influenced by some socio-demographic aspects of patients, such as gender, suggesting that women may be less inclined than men to seek antibiotics. This result is noteworthy in light of previous research indicating that women typically receive a higher number of antibiotic prescriptions compared to men [60–63]. Interpreting our findings within this broader context may highlight gender-related differences in prescribing trends that might be driven by clinical factors rather than by relational dynamics such as requesting behavior. Also, the educational level has an influence on patients’ intention to ask for antibiotics with a higher academic degree, having completed a bachelor’s and having professional training, significantly lowering the intention to ask for antibiotics. All these conditions imply the completion of education beyond the mandatory level. The results align with existing literature on antibiotic consumption, which indicates that higher educational attainment is associated with greater health literacy and therefore a more informed and cautious approach to antibiotic use [64–66].

One main limitation of our study is the small overall sample. Future research should involve a larger number of participants to be able to apply a statistical description of the interaction terms. Moreover, the low rate of participating doctors could have introduced a selection bias in the process of the selection of the patients.

The data collection was done after consultations with the doctor, in which antibiotics were not prescribed. This specific context may have elevated the reported trust levels and influenced participants stated intentions to request antibiotics. Furthermore, the predominance of older adults within the sample restricts the generalizability of the findings to broader populations. Another limitation is self-level measurement, which may deflate, especially in evaluating trust. Moreover, the outcome was the intention to request antibiotics as proposed in a hypothetical scenario. Future research should measure actual intention or behavior. Finally, one limitation of this study is that the measure of doctors’ trust asks doctors to evaluate their level of trust in their patients, not patients’ perceptions of doctor’s trust. Our use of this scale assumes that patients are sensitive to relational cues during consultations and that perceived trust may influence their communication choices. Future research could benefit from including direct measures of patients’ awareness of their doctor’s trust.

This study represents a first step toward a more dyadic approach in research on patient-provider interactions. Future studies could build on these findings by examining the interaction as it unfolds in real time. Medical visits are dynamic processes in which both participants’ positions may shift and evolve during the encounter, shaped by the interaction itself. For example, it could be interesting to verify whether the patient’s request for antibiotics, and the doctor’s willingness to prescribe an antibiotic, changes from the first stage of the visit, to immediately after the examination, and at the end of the visit. A qualitative triangulated approach, such as observations conducted by an external observer, could offer a more objective way to assess the medical rationale guiding doctors’ decisions and how patients signal their needs and intentions.

## Conclusion

One of the most important strengths of this study is the novelty brought by the approach considering reciprocity in doctor-patient relationships and the analysis of doctors’ influence on patients’ behavior. Also, the analysis, performed with the Generalized Estimation Equations method, allowed us to generate results that consider the grouping of patients and, therefore, the non-independence of data. This study contributes to the growing body of research on antibiotic stewardship by offering a more comprehensive and dyadic understanding of the interpersonal dynamics that shape patients’ predisposition to request antibiotics.

The findings suggest that interventions aimed at reducing inappropriate antibiotic use may benefit from addressing the relational dynamics embedded in medical encounters. Training programs that support physicians in fostering trust while maintaining professional authority could help mitigate unnecessary antibiotic demand. Moreover, the moderating role of physicians’ trust in patients, particularly among individuals who tend to experience more severe symptoms or higher levels of concern when sick, underscores the importance of tailored communication strategies during clinical consultations.

Socio-demographic patterns observed in the data further reveal disparities in the general inclination to ask for antibiotic treatment, with women and more highly educated individuals reporting lower intentions to request antibiotics when presented with a hypothetical scenario. These insights point to the relevance of demographic targeting in public health campaigns and suggest that improving health literacy across different social strata may be an effective approach to promoting responsible antibiotic use on a broader scale.

Together, these results emphasize that trust in the medical encounter is not unidirectional but reciprocal, and that physicians’ perceptions play a critical role in shaping patient behavior. A deeper consideration of this bidirectional trust dynamic can inform both clinical practice and health policy, ultimately contributing to more effective strategies for antibiotic stewardship.

## Electronic supplementary material

Below is the link to the electronic supplementary material.

Supplementary Material 1

## Data Availability

The dataset can be shared with other researchers upon reasonable request to the authors.

## Footnotes

1 Note: “paskint” = intention of patients to ask for antibiotics; “page” = patients’ age; “pgender” = patient gender; “pedu” = patient’s higher educational degree obtained; “phealth” = patient’s evaluation of health status; “pchronic” = presentation of chronic illnesses; “pconcern” = patient rated level of concern; “psusceptibility” = patient perceived susceptility to illness; “psev1” = patient perceived severity of symptoms of cold and flu; “psev2” = patient perceived severity of symptoms of urinary or respiratory infection; “ptrust” = patient rated trust in the visiting doctor; “dtrust1” = doctor rated abilities of patients of communicating; “dtrust2” = doctor rated respect of patient of professional boundaries.

2 Note: as for the previous equation. dtrust1ij×psusceptibilityij is an example of an interaction term where i represents the groups (8 doctors), j the individuals within the groups, and ϵij represents the random error. Variables starting with “p” are measured at the patient level, and those starting with “d” are measured at the doctor level.

## Declarations

### Disclaimer

All the data in tables and figures was gathered through the “COMETA” project procedure in the Ticino region of Switzerland, from May to July 2024, and was analyzed in the Università della Svizzera Italiana (Switzerland) from September to October 2024.

## Abbreviations

AMR: antimicrobial resistance
ESRC: Economic and Social Research Council
GEE: Generalized Estimating Equations
QIC: Quasi-likelihood under the Independence model Criterion
RTI: Respiratory Tract Infection
