# Supplementary material for: A cross-sectional study examining the role of doctors’ trust in patients’ requests for antibiotics: a neglected perspective
Source: Arch Public Health. 2025 Jul 17;83:188. doi: 10.1186/s13690-025-01677-2 (PMC12269201; doi:10.1186/s13690-025-01677-2)
Supplement: Supplementary file 1 — Supplementary Material 1 [file 13690_2025_1677_MOESM1_ESM.docx]

**A cross-sectional study Examining the role of doctors’ trust in patients’ requests for antibiotics: a neglected perspective (supplementary material)**

**Script.**

Script for the Generalized Estimating Equations

#Installing packages

# install.packages("geepack")

install.packages("haven")

library(haven)

library(geepack)

# Ensure mgroups is a a factor (clustering variable)

dataset$clusters <- as.factor(dataset$clusters)

levels(dataset$clusters)

dataset$mgroups <- as.factor(dataset$mgroups)

levels(dataset$mgroups)

# Formatting demographic variables (nominal)

dataset$pgender <- as.factor(dataset$pgender)

levels(dataset$pgender) <- c("Maschio", "Femmina")

levels(dataset$pgender)

dataset$pchronic <- as.factor(dataset$pchronic)

levels(dataset$pchronic) <- c("No", "Sí", "Preferisco non specificarlo")

levels(dataset$pchronic)

table(dataset$pchronic)

dataset$pchronic[dataset$pchronic == "Preferisco non specificarlo"] <- NA

table(dataset$pchronic)

dataset$pchronic <-droplevels(dataset$pchronic)

table(dataset$pchronic)

dataset$pedu <- as.factor(dataset$pedu)

levels(dataset$pedu) <- c("Scuole dell'obbligo", "Formazione professionale", "Scuola superiore", "Bachelor", "Titolo post-laurea")

levels(dataset$pedu)

dataset$phealth <- as.factor(dataset$phealth)

levels(dataset$phealth) <- c("Molto scadente", "Scadente", "Nella media", "Buono", "Molto buono")

levels(dataset$phealth)

#checking missing data

dataset_clean <- dataset[complete.cases(dataset[, c("askint", "page", "pgender", "pedu", "phealth", "pchronic", "pworry", "risk", "psev1", "psev2", "ptrust", "mtrust1", "mtrust2")]), ]

# Specify the Generalized Estimating Equations (GEE) model

model_formula <- askint ~ page + pgender + pedu + phealth + pchronic + pworry + risk + psev1 + psev2 + ptrust + mtrust1 + mtrust2

# Fit the GEE model

gee_model <- geeglm(formula = model_formula, data = dataset_clean, id = clusters, family = gaussian(link = "identity"), corstr = "exchangeable")

summary(gee_model)

# mtrust1*pworry

model_formula_mtrust1_pworry <- askint ~ page + pgender + pedu + phealth + pchronic + pworry + risk + psev1 + psev2 + ptrust + mtrust1 + mtrust2 + mtrust1*pworry

gee_model_mtrust1_pworry <- geeglm(formula = model_formula_mtrust1_pworry, data = dataset_clean, id = clusters, family = gaussian(link = "identity"), corstr = "exchangeable")

summary(gee_model_mtrust1_pworry)

# mtrust1*risk

model_formula_mtrust1_risk <- askint ~ page + pgender + pedu + phealth + pchronic + pworry + risk + psev1 + psev2 + ptrust + mtrust1 + mtrust2 + mtrust1*risk

gee_model_mtrust1_risk <- geeglm(formula = model_formula_mtrust1_risk, data = dataset_clean, id = clusters, family = gaussian(link = "identity"), corstr = "exchangeable")

summary(gee_model_mtrust1_risk)

# mtrust1*psev1

model_formula_mtrust1_psev1 <- askint ~ page + pgender + pedu + phealth + pchronic + pworry + risk + psev1 + psev2 + ptrust + mtrust1 + mtrust2 + mtrust1*psev1

gee_model_mtrust1_psev1 <- geeglm(formula = model_formula_mtrust1_psev1, data = dataset_clean, id = mgroups, family = gaussian(link = "identity"), corstr = "exchangeable")

summary(gee_model_mtrust1_psev1)

# mtrust1*psev2

model_formula_mtrust1_psev2 <- askint ~ page + pgender + pedu + phealth + pchronic + pworry + risk + psev1 + psev2 + ptrust + mtrust1 + mtrust2 + mtrust1*psev2

gee_model_mtrust1_psev2 <- geeglm(formula = model_formula_mtrust1_psev2, data = dataset_clean, id = mgroups, family = gaussian(link = "identity"), corstr = "exchangeable")

summary(gee_model_mtrust1_psev2)

# mtrust1*ptrust

model_formula_mtrust1_ptrust <- askint ~ page + pgender + pedu + phealth + pchronic + pworry + risk + psev1 + psev2 + ptrust + mtrust1 + mtrust2 + mtrust1*ptrust

gee_model_mtrust1_ptrust <- geeglm(formula = model_formula_mtrust1_ptrust, data = dataset_clean, id = mgroups, family = gaussian(link = "identity"), corstr = "exchangeable")

summary(gee_model_mtrust1_ptrust)

# mtrust2*pworry

model_formula_mtrust2_pworry <- askint ~ page + pgender + pedu + phealth + pchronic + pworry + risk + psev1 + psev2 + ptrust + mtrust1 + mtrust2 + mtrust2*pworry

gee_model_mtrust2_pworry <- geeglm(formula = model_formula_mtrust2_pworry, data = dataset_clean, id = mgroups, family = gaussian(link = "identity"), corstr = "exchangeable")

summary(gee_model_mtrust2_pworry)

# mtrust2*risk

model_formula_mtrust2_risk <- askint ~ page + pgender + pedu + phealth + pchronic + pworry + risk + psev1 + psev2 + ptrust + mtrust1 + mtrust2 + mtrust2*risk

gee_model_mtrust2_risk <- geeglm(formula = model_formula_mtrust2_risk, data = dataset_clean, id = mgroups, family = gaussian(link = "identity"), corstr = "exchangeable")

summary(gee_model_mtrust2_risk)

# mtrust2*psev1

model_formula_mtrust2_psev1 <- askint ~ page + pgender + pedu + phealth + pchronic + pworry + risk + psev1 + psev2 + ptrust + mtrust1 + mtrust2 + mtrust2*psev1

gee_model_mtrust2_psev1 <- geeglm(formula = model_formula_mtrust2_psev1, data = dataset_clean, id = mgroups, family = gaussian(link = "identity"), corstr = "exchangeable")

summary(gee_model_mtrust2_psev1)

# mtrust2*psev2

model_formula_mtrust2_psev2 <- askint ~ page + pgender + pedu + phealth + pchronic + pworry + risk + psev1 + psev2 + ptrust + mtrust1 + mtrust2 + mtrust2*psev2

gee_model_mtrust2_psev2 <- geeglm(formula = model_formula_mtrust2_psev2, data = dataset_clean, id = mgroups, family = gaussian(link = "identity"), corstr = "exchangeable")

summary(gee_model_mtrust2_psev2)

# mtrust2*ptrust

model_formula_mtrust2_ptrust <- askint ~ page + pgender + pedu + phealth + pchronic + pworry + risk + psev1 + psev2 + ptrust + mtrust1 + mtrust2 + mtrust2*ptrust

gee_model_mtrust2_ptrust <- geeglm(formula = model_formula_mtrust2_ptrust, data = dataset_clean, id = clusters, family = gaussian(link = "identity"), corstr = "exchangeable")

summary(gee_model_mtrust2_ptrust)

# Testing model fit (only significant interactions)

QIC(gee_model)

QIC(gee_model_mtrust2_risk)

QIC(gee_model_mtrust1_pworry)

QIC(gee_model_mtrust2_psev1)

QIC(gee_model_mtrust2_psev2)

# creating plot models for interactions

library(interactions)

mean_mtrust1 <- mean(dataset_clean$mtrust1)

sd_mtrust1 <- sd(dataset_clean$mtrust1)

mean_mtrust2 <- mean(dataset_clean$mtrust2)

sd_mtrust2 <- sd(dataset_clean$mtrust2)

# Creating modx.values because 1 SD above the mean excedes the values measured on the scale

modx_values_mtrust2 <- c(mean_mtrust2 - 0.7 * sd_mtrust2, mean_mtrust2, mean_mtrust2 + 0.7 * sd_mtrust2)

modx_values_mtrust1 <- c(mean_mtrust1 - 0.7 * sd_mtrust1, mean_mtrust1, mean_mtrust1 + 0.7 * sd_mtrust1)

# Creating labels

modx_labels <- c("- 0.7 SD", "Mean", "+ 0.7 SD")

# mtrust1*pworry

interact_plot(gee_model_mtrust1_pworry, pred = pworry, modx = mtrust1, plot.points = TRUE, modx.values = c (mean_mtrust1 + sd_mtrust1, mean_mtrust1, mean_mtrust1 - sd_mtrust1), xlim = c(1,5), )

interact_plot(gee_model_mtrust1_pworry, pred = pworry, modx = mtrust1, modx.values = modx_values_mtrust1, modx.labels = modx_labels, plot.points = TRUE)

# mtrust2*risk

interact_plot(gee_model_mtrust2_risk, pred = risk, modx = mtrust2, modx.values = modx_values_mtrust2, modx.labels = modx_labels, plot.points = TRUE)

# mtrust2*psev1

interact_plot(gee_model_mtrust2_psev1, pred = psev1, modx = mtrust2, modx.values = modx_values_mtrust2, modx.labels = modx_labels, plot.points = TRUE)

# mtrust2*psev2

interact_plot(gee_model_mtrust2_psev2, pred = psev2, modx = mtrust2, modx.values = modx_values_mtrust2, modx.labels = modx_labels, plot.points = TRUE)
